# Genome Assemblies across the Diverse Evolutionary Spectrum of *Leishmania* Protozoan Parasites

**DOI:** 10.1128/MRA.00545-21

**Published:** 2021-09-02

**Authors:** Wesley C. Warren, Natalia S. Akopyants, Deborah E. Dobson, Christiane Hertz-Fowler, Lon-Fye Lye, Peter J. Myler, Gowthaman Ramasamy, Achchuthan Shanmugasundram, Fatima Silva-Franco, Sascha Steinbiss, Chad Tomlinson, Richard K. Wilson, Stephen M. Beverley

**Affiliations:** a Department of Animal Sciences, Institute of Data Science and Informatics, Bond Life Sciences Center, University of Missouri, Columbia, Missouri, USA; b Department of Molecular Microbiology, Washington University School of Medicine, St. Louis, Missouri, USA; c Institute of Integrative Biology, University of Liverpool, Liverpool, United Kingdom; d Center for Global Infectious Disease Research, Seattle Children’s Research Institute, Seattle, Washington, USA; e Department of Pediatrics, University of Washington, Seattle, Washington, USA; f Department of Biomedical Informatics and Medical Education, University of Washington, Seattle, Washington, USA; g Department of Global Health, University of Washington, Seattle, Washington, USA; h Wellcome Sanger Institute, Hinxton, Cambridgeshire, United Kingdom; i McDonnell Genome Institute, Washington University School of Medicine, St. Louis, Missouri, USA; j The Steve and Cindy Rasmussen Institute for Genomic Medicine, Nationwide Children’s Hospital, Columbus, Ohio, USA; University of Maryland School of Medicine

## Abstract

We report the high-quality draft assemblies and gene annotations for 13 species and/or strains of the protozoan parasite genera *Leishmania*, *Endotrypanum*, and *Crithidia*, which span the phylogenetic diversity of the subfamily Leishmaniinae within the kinetoplastid order of the phylum Euglenazoa. These resources will support studies on the origins of parasitism.

## ANNOUNCEMENT

*Leishmania* species are widespread parasites of mammals transmitted by biting insects. Over 1.7 billion people worldwide are at risk, with hundreds of millions of people infected (1–4). The genus comprises more than 50 species, which are primarily zoonotic but in humans cause disease ranging from mild cutaneous lesions to more disseminated forms to fatal visceralizing disease (5). While parasitism by *Leishmania* has been intensively studied, the species-specific factors that enable mammalian or insect host infections are less well understood. To provide a broad phylogenetic snapshot, we selected a spectrum of species and strains across the subfamily Leishmaniinae (5), targeting lineages within the subgenera *Leishmania*, *Viannia*, and *Mundinia*, as well as the allied genus *Endotrypanum* and the outgroup Crithidia fasciculata (Table 1). The WU Institutional Biosafety Committee reviewed and approved the parasite work reported here (01-015).

**TABLE 1:** Description of *Leishmania* species and strains, including assembly parameters and links

Genus or subgenus	Species	Strain	WHO code	Source	Provenance	BioProject accession no.^*a*^	Sequencing platforms	Assembler	No. of contigs	*N*_50_ (bp)	Assembly size (bp)	% GC	No. of pseudochromosomes	No. of protein-coding genes	Reference for other assembly
*L. Leishmania*	L. major	SD75.1 (clone)	MHOM/SN/74/SD	Human, cutaneous	D. Sacks, Bethesda, MD	PRJNA50303	454, Illumina	Newbler	891	95,380	31,727,271	59.3	36	8,818	
		LV39 (clone 5)	MHOM/Sv/59/P	Human, cutaneous	R. Titus, Boston, MA	PRJNA50301	454, Illumina	Newbler	1,667	71,452	31,961,985	59.5	36	8,971	
	*L. gerbilli*	LEM452	MRHO/CN/60/GERBILLI	Gerbil	P. Bastien, J.-P. Dedet, Montpellier, France	PRJNA192717	454, Illumina	AllPaths	1,248	57,008	30,822,621	59.6	36	8,599	
	*L. turanica*	LEM423	MRHO/SU/65/VL	Gerbil	P. Bastien, J.-P. Dedet, Montpellier, France	PRJNA192712	454, Illumina	AllPaths	1,669	39,210	30,876,294	59.5	36	8,608	
	*L. arabica*	LEM1108	MPSA/SA/83/JISH220	*Psammomys* sp.	P. Bastien, J.-P. Dedet, Montpellier, France	PRJNA192710	454, Illumina	AllPaths	1,530	52,119	30,774,332	59.2	36	8,646	
	*L. tropica*	L590	MHOM/IL/1990/P283	Human, cutaneous	C. Jaffe, Jerusalem, Israel	PRJNA169676	454, Illumina	AllPaths	1,938	32,739	31,326,083	59.6	36	8,824	12
	*L. aethiopica*	L147	MHOM/ET/1972/L100	Human, diffuse cutaneous, relapsing	C. Jaffe, Jerusalem, Israel	PRJNA169673	454, Illumina	AllPaths	1,758	38,498	31,026,739	60.1	36	8,722	
*L. Viannia*	*L. braziliensis*	M2903	MHOM/BR/75/M2903	Human, cutaneous	J. Shaw, Brazil	PRJNA165955	454, Illumina	Newbler	3,934	61,918	32,590,753	57.4	35	9,269	
	L. panamensis	L13	MHOM/COL/81/L13	Human, mucosal	N. Saravia, Cali, Colombia	PRJNA165959	454, Illumina	AllPaths	3,163	22,576	31,108,242	57.4	35	8,665	
*L. Mundinia*	*L. enriettii*	LEM3045	MCAV/BR/95/CUR3	Cavia porcellus (guinea pig)	P. Bastien, J.-P. Dedet, Montpellier, France	PRJNA192711	454, Illumina	AllPaths	1,171	102,666	30,427,298	59.3	36	8,731	14
	*L. martiniquensis*	LEM2494	MHOM/MQ/92/MAR1	Human, diffuse cutaneous, HIV	P. Bastien, J.-P. Dedet, Montpellier, France	PRJNA192703	454, Illumina	AllPaths	628	147,290	30,528,357	59.6	36	8,483	14
*Endotrypanum*	*Endotrypanum monterogei*	LV88	None	*Choloepus hoffmanni* (sloth)	Michael Chance, Liverpool, UK	PRJNA165953	454, Illumina	Newbler	3,517	33,059	32,086,870	52.5	36	8,285	13
*Crithidia*	*Crithidia fasciculata*	Cf-C1 (clone)	None	Mosquito	Larry Simpson, Los Angeles, CA	PRJNA165885	454, Illumina, PacBio	HGAP	494	778,443	41,297,378	57.0	30	9,619	8

a Each BioProject link contains links to the current assembly, primary data sets, and other relevant information.

Parasites were cultivated in M199 or Schneider’s medium (6) and grown to late log phase before harvesting, lysis, and DNA purification by phenol-chloroform extraction and/or banding in CsCl gradients (to remove mitochondrial maxi- or minicircle DNA). Sequencing libraries were generated using the Illumina paired-end DNA sample preparation kit (PE-102-1001) according to the manufacturer’s directions. Fragment libraries of 3 and 8 kb were prepared using protocols for 454 sequencing (Roche Life Sciences). Sequencing was performed on either a 454 GS FLX Titanium (average read length, 305 bp; Roche 454 Life Sciences) or Illumina Genome Analyzer IIx (GAIIx) and HiSeq 2000 instruments (paired-end 100-bp format), except for *Crithidia*, which additionally utilized long reads generated on an RS II instrument (P5/C3 chemistry; Pacific Biosciences) (7). The total sequence genome coverage on the Illumina GAIIx and HiSeq 2000 instruments was on average 105× with tiered library insert sizes (50× fragments; 45 × 3 kb, 10 × 8 kb, and 0.05 × 40 kb). For all Illumina sequences, we used the read processing steps within the AllPaths-LG (8) software prior to *de novo* assembly, which incorporates read error correction methods described by Pevzner et al. (9). Genome assemblies were conducted with default parameters using Newbler v2.0.1 (10) for 454 reads, AllPaths-LG (8) for Illumina reads, and HGAP v3 (11) for long reads (Table 1). Contigs and scaffolds were organized into pseudochromosomes using ABACAS2 (https://github.com/satta/ABACAS2), a successor to ABACAS (12), by alignment with the Leishmania major Friedlin genome sequence, with the exception of Leishmania braziliensis M2903 and Leishmania panamensis, which were aligned to the *L. braziliensis* M2904 genome. The estimated haploid genome sizes ranged from 30.4 to 41.3 megabases (13).

Gene annotations were performed using the comprehensive Companion tool, which incorporates a variety of *de novo* prediction criteria, as well as information from closely related genomes when available (14). The number of protein-coding genes predicted ranged from 8,285 to 9,619, typical of other *Leishmania* species (13). Full annotations, as well as a variety of tools for the visualization or analysis of these genomes, are available from TriTrypDB (www.tritrypdb.org).

### Data availability.

The assemblies have been deposited in the NCBI GenBank repository under the BioProject accession numbers in Table 1, including links to the primary data and annotations (PRJNA50303, PRJNA50301, PRJNA192717, PRJNA192712, PRJNA192710, PRJNA169676, PRJNA169673, PRJNA165955, PRJNA165959, PRJNA192711, PRJNA192703, PRJNA165953, and PRJNA165885). The chromosome builds are available through the TriTrypDB portal (http://tritrypdb.org/tritrypdb).
